# Dr. Richard L. Stoddard

**Published:** 1918-07

**Authors:** 


**Dr. Richard L. Stoddard** of Rochester died April 29, age 48. He graduated from the Albany Medical College in 1895.

